# A Scoping Review Protocol of Social Determinants of HIV/TB Coinfections in Sub-Saharan Africa

**DOI:** 10.3390/mps7010004

**Published:** 2024-01-04

**Authors:** Lucas Banda, Olanrewaju Oladimeji

**Affiliations:** 1 Department of Public Health, Faculty of Medicine and Health Sciences, Walter Sisulu University, Mthatha 5099, South Africa; lbanda@wsu.ac.za; 2 Department of Epidemiology and Biostatistics, School of Public Health, Sefako Makgatho Health Sciences University, Pretoria 0208, South Africa

**Keywords:** Human Immunodeficiency Virus (HIV), tuberculosis (TB), comorbidity, cooccurrence, social determinants, risk factors, sub-Saharan Africa

## Abstract

**Introduction:** Tuberculosis (TB) and Human Immunodeficiency Virus (HIV) remain major public health issues in sub-Saharan Africa. The co-occurrence of these diseases is a growing concern in the region, and social determinants, the circumstances under which people are born, live, work, and age, are known to influence the risk of disease transmission, diagnosis, treatment, and outcomes. Here, we present a protocol for the evidence synthesis on the social determinants of HIV/TB coinfections in sub-Saharan Africa. The high prevalence of Tuberculosis (TB) and Human Immunodeficiency Virus (HIV) in sub-Saharan Africa presents significant public health challenges. TB/HIV comorbidity is influenced by various social determinants, including social, economic, cultural, and environmental factors, impacting disease transmission risk, accurate diagnosis, and treatment outcomes. This study protocol aims to provide an evidence synthesis on the social determinants of HIV/TB coinfection in sub-Saharan Africa. **Methods and analysis:** The researchers will use the Arksey and O’Malley’s (2005) methodological framework to guide the scoping review. First, databases such as PubMed, MEDLINE, Web of Science, and PsychInfo will be searched. The researchers will then proceed in two steps. Before finalising the study selection, two independent reviewers will examine the article titles and abstracts for eligibility and inclusion. The researchers will then conduct a full-text screening of the articles based on the selected titles and abstracts. The authors’ tool will be used to extract data, ensuring that the articles are properly screened and that the risk of bias is minimized. The chosen studies will be examined using a standardized tool to examine all bibliographic data and study characteristics. **Ethics and dissemination:** The review will provide an overview of the social determinants influencing the prevalence and outcomes of TB/HIV comorbidity in the region, as well as identify any research gaps. Policymakers, researchers, and healthcare professionals will benefit from the findings in developing targeted interventions to address the social determinants of TB/HIV comorbidity in sub-Saharan Africa.

## 1. Introduction

Sub-Saharan Africa faces serious public health challenges due to the high prevalence of Tuberculosis and Human Immunodeficiency Virus. The co-occurrence of these diseases, known as TB/HIV comorbidity, is of markedly great concern, being influenced by various social determinants [1,2]. TB and HIV are not merely health issues but are also deeply interconnected with social determinants of health. The social determinants of health, the circumstances under which people are born, live, work, and age [3], comprise a broad range of social, economic, cultural, and environmental factors that contribute to an individual’s or group’s health status [4,5,6]. These determinants can have an extensive impact on the risk of disease transmission, the chances of accurate and timely diagnosis, and the outcomes of treatment strategies [4,7]. Socioeconomic status, living conditions, cultural beliefs, and access to healthcare services can have a significant impact on the risk, prevalence, and treatment outcomes of TB/HIV comorbidity in this region.

The purpose of this study is to conduct a comprehensive exploration of how various socio-environmental factors contribute to the prevalence and impacts of TB/HIV comorbidity in sub-Saharan Africa. It will focus on analyzing and understanding complicated interactions between economic status, cultural beliefs, environmental conditions, accessibility to healthcare services, and the rate of TB/HIV coinfections. By focusing on sub-Saharan Africa, the study will also reveal the distinctive regional differences present in the disease prevalence to understand the underlying determinants causing these disparities. Additionally, cultural and behavioral aspects unique to this region and their effect on disease transmission and outcomes will also be scrutinized. Overall, this study aims to provide a holistic understanding of not only the prevalence and handling of TB/HIV comorbidity in sub-Saharan Africa but also its determinants.

STRENGTHS AND LIMITATIONS OF THIS STUDY

⮚ This scoping review will provide a comprehensive overview of the social determinants of TB/HIV comorbidity in sub-Saharan Africa, which can help identify research gaps and guide future research directions.
⮚ The review will follow a well-established scoping review methodology that provides a rigorous and transparent approach to evidence synthesis.
⮚ The use of multiple electronic databases and a comprehensive search strategy will increase the likelihood of capturing relevant studies and reducing the potential for bias in the selection of studies.
⮚ The study will provide valuable insights for policymakers, researchers, and healthcare professionals in developing targeted interventions to address the social determinants of TB/HIV comorbidity in sub-Saharan Africa.
⮚ To the best of our knowledge, this will be the first study to review social determinants of health among TB/HIV-coinfected patients in SSA, and the results will be useful given the high burden of TB/HIV infections and related deaths in SSA.
⮚ The review will only include studies published in English, which may exclude relevant studies published in other languages. The review will only cover studies published in the last decade (2014–2022), which may exclude older studies that could be relevant.
⮚ The scoping review methodology does not involve a critical appraisal of the quality of the studies included, which may affect the reliability of the findings. The review may be limited by the availability and quality of the data reported in the included studies, which may impact the accuracy and completeness of the findings. It is possible that our search strategy will miss relevant studies; we will mitigate this risk by searching multiple databases and manually searching review articles and meta-analyses.

## 2. Method

The scoping review will follow the framework outlined by Arksey and O’Malley and the Joanna Briggs Institute (JBI) scoping review methodology [8,9]. The review will include the following stages. The review process consists of 5 stages, as shown in Table 1.

*Stage 1—Identifying the research questions*

To develop research questions, a preliminary exploration of literature was conducted. The primary research question focuses on describing how social determinants of health may influence TB/HIV comorbidity. Subsequent to the primary research question are several research objectives, specifically:

1. To identify the social determinants of health that contribute to the comorbidity of TB/HIV comorbidity in sub-Saharan Africa.
2. To describe the influence of environmental factors on the occurrence of TB/HIV comorbidity in sub-Saharan Africa.
3. To explore the relationship between social determinants of health and access to TB/HIV prevention, treatment, and care in sub-Saharan Africa.
4. To identify the disparities in TB/HIV comorbidity across different demographic groups and geographical regions in sub-Saharan Africa.
5. To describe the impact of cultural and behavioral factors on the prevalence and transmission of TB/HIV comorbidity in sub-Saharan Africa.

The research question for this scoping review is, what are the social determinants of TB/HIV comorbidity that influence prevalence and outcome in sub-Saharan Africa?

*Stage 2—Identifying relevant studies*

The study’s review will have as information sources both peer-reviewed journal articles and gray literature. Systematic search queries will be used for both groups using university online library resources. PubMed, MEDLINE, Web of Science, PsychInfo, and Google Scholar will be among the electronic databases utilized. Additionally, the reference lists of the included studies will be scrutinized to uncover any relevant studies. Gray literature databases such as Open Grey and Grey Literature Report will also be searched to unearth studies and reports pertinent to this review. The targeted keywords for our search strategy will be ‘HIV/AIDS’, ‘tuberculosis’, ‘comorbidity’, ‘Sub-Saharan Africa’, ‘adults’, and specific social determinants such as ‘education’, ‘economic status’, ‘environment’, ‘conflict’, ‘food insecurity’, and ‘healthcare access’. The search will be limited to studies published in English from the last decade (2014–2022). To give a balanced picture of the available evidence, an extensive and systematic gray literature search will be performed. Table 2 shows the source of gray literature and provides a commentary on how each source will be searched.

*Search String*

Table 3 provides a comprehensive outline of the search strategy. The lead researcher, in conjunction with the research team and an academic librarian, will determine pertinent search terms. Keywords such as Human Immunodeficiency Virus (HIV), Tuberculosis (TB), comorbidity, social determinants, risk factors, sub-Saharan Africa, and 18 years and above will be used and searched as keywords in the title, abstract, and subject headings (i.e., MeSH terms). The review articles retrieved will be screened for their titles, abstracts, and index terms. Articles from each database will be imported into Zotero 6.0.30, a reference management software program, for record keeping, article tracking, and reference list creation, which will be incorporated into the final report.

## 3. Synthesis of Eligibility Criteria

Table 4 features all the peer-reviewed, published studies that outline the social determinants of TB/HIV comorbidity in sub-Saharan Africa, encompassing the following inclusion criteria: (i) published in English; (ii) within the timeframe of 2014–2022; (iii) conducted in any setting; and (iv) consisting of randomized controlled trials, intervention studies (including quasi-experimental designs), qualitative and quantitative studies, and (v) examining HIV/TB comorbidity patients as the study population. Any studies that fail to meet the criteria will be excluded.

## 4. Study Design

All primary peer-reviewed study designs will be considered in this review, comprising qualitative and quantitative studies. On the other hand, systematic reviews, meta-analyses, and review articles will be excluded.

## 5. Setting

This review will encompass studies that addressed the social determinants of HIV/TB co-occurrence in sub-Saharan Africa.

*Stage 3—Study selection*

**Figure 1:**
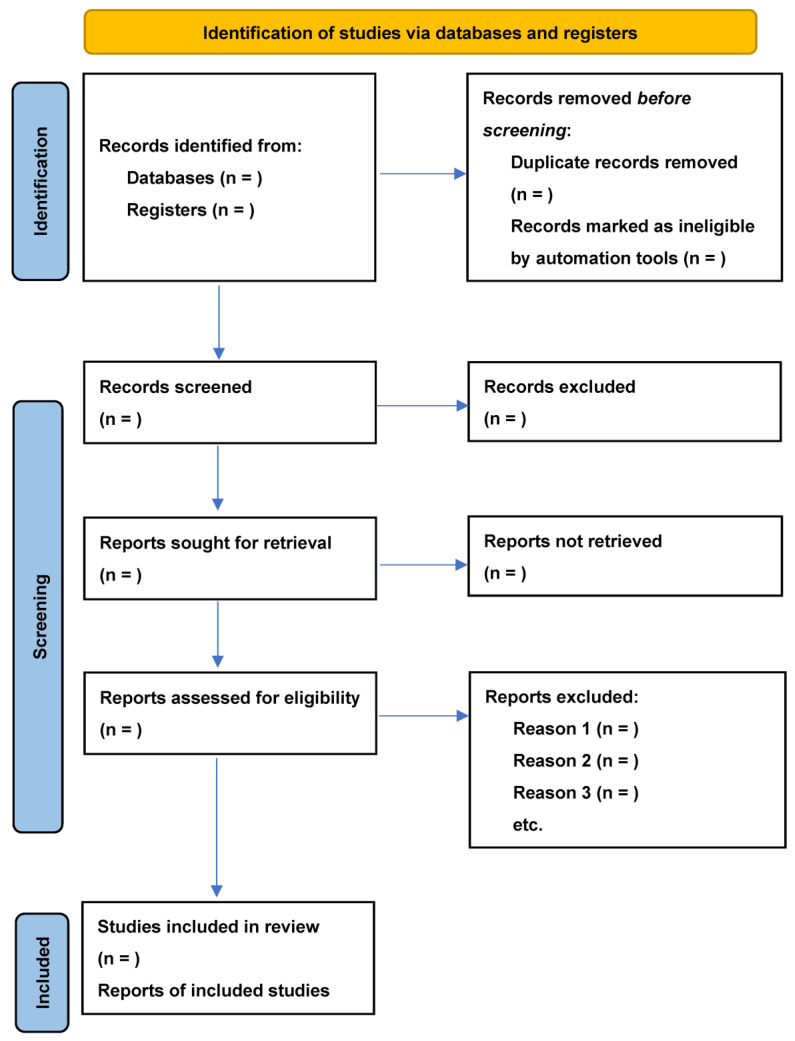
PRISMA flowchart [11].

For this scoping review, two reviewers will individually scrutinize the titles and abstracts of the identified studies. The inclusion criteria are studies examining the social determinants of TB/HIV co-occurrence in sub-Saharan Africa, published in English, and with full-text availability. In the event of discrepancies between the reviewers, they will convene and reach a mutual agreement via discussion. The article screening process for this review will comprise two stages. Firstly, two team members will independently assess the titles and abstracts of all retrieved citations for eligibility against the established inclusion criteria (Table 3). If relevant meta-analyses or reviews are identified, we will scan their reference lists. To ensure that the eligibility criteria are uniformly applied, two team members will conduct an initial independent screening of a sample of retrieved articles. Articles deemed relevant by one or both reviewers will be included in the full-text review. The inter-rater reliability will be evaluated using the percentage agreement. If the percentage agreement is less than 80%, the inclusion and exclusion criteria will be clarified. The second stage will involve deduplication of different databases and a team member screening the titles and abstracts of the remaining articles to exclude those that do not meet the eligibility criteria. The titles and abstracts that satisfy the inclusion criteria will be reviewed in full. Any disagreements regarding eligibility will be resolved through discussion among reviewers or with the intervention of a third party, if necessary. The study selection process will be documented using the Preferred Reporting Items for Systematic Reviews and Meta-Analyses (PRISMA) flowchart (Figure 1).

*Stage 4—Charting the data*

Two reviewers will use a standardized data extraction form to independently extract data from the included studies. The form will cover the author and year of publication, study design, study setting, sample size, social determinants investigated, and main findings. Relevant information will be collected and sorted from abstracts of selected articles, including study title, objectives, method, sample size, barriers, and strategic facilitators. A data extraction framework was developed to guide the eligible full text retrieved in the literature, based on the initial scoping phase. For each article, the table will include information on HIV/TB comorbidity determinants, characteristics of the study population, study method, sample size, and the type of determinants. To ensure consistency, two team members will pilot-test a sample of the included studies and modify categories if new ones emerge. Any disagreements will be resolved through team consultations, and authors of eligible abstracts with missing information will be contacted. The team members piloting the framework will be responsible for independently charting the data from each of the included studies. Any disagreements in the data extracted by the two team members will be discussed until a consensus is reached or settled by arbitration of a third reviewer, if required.

*Stage 5—Collating, summarizing, and reporting the findings*

This scoping review will utilize a narrative synthesis approach to summarize the existing knowledge on social determinants of HIV/TB comorbidity in sub-Saharan Africa and propose effective strategies to reduce the incidence and mortality rates in this population. The review will present a thematic narrative of the various determinants identified from the selected studies and add to the body of knowledge by summarizing, interpreting, and reporting on the determinants using a standardized tool. Additionally, the scoping review will identify gaps in the existing literature and suggest future research topics. Rather than evaluating individual study quality, the report will provide an overview of the available evidence, aggregate results, and present a summary outline. The findings of the scoping review will be used to develop targeted strategies to better understand the complex factors that contribute to the occurrence and treatment of TB/HIV comorbidity, given its high prevalence. Because the review will be based on publicly available publications and materials, no ethical approval will be required. The study’s findings will be peer-reviewed and published in a scientific journal, and the abstract will be presented at local and international conferences. The review’s findings will be distributed to health ministries, TB/HIV-led community-based organizations, and policymakers in order to inform policy direction on the determinants of TB/HIV health outcomes. The review will provide a summary of the determinants’ barriers and strategic facilitators.

## 6. Limitations

The review will only include studies published in English, which may exclude relevant studies published in other languages. The review will only cover studies published in the last decade (2014–2022), which may exclude older studies that could be relevant.

The scoping review methodology does not involve a critical appraisal of the quality of the studies included, which may affect the reliability of the findings. The review may be limited by the availability and quality of the data reported in the included studies, which may impact the accuracy and completeness of the findings. It is possible that our search strategy will miss relevant studies; we will mitigate this risk by searching multiple databases and manually searching review articles and meta-analyses.

## 7. Conclusions and Expected Policy Implication

The purpose of this scoping review is to provide an overview of the social determinants of tuberculosis/HIV co-morbidity in sub-Saharan Africa. The review will synthesize existing literature to map the social determinants of TB/HIV comorbidity in the region. This scoping review’s findings will be useful to policymakers, researchers, and healthcare professionals in developing targeted interventions to address the social determinants of TB/HIV comorbidity in sub-Saharan Africa.

## Figures and Tables

**Table 1:** Scoping review protocol design, adapted from the Arksey and O’Malley framework with Prisma-ScR.

Arksey and O’Malley Framework [8]	PRISMA-ScR [10] Protocol Relevant Checklist Items
1. Identify the research questions	Objectives
2. Identifying relevant studies	Information sources, eligibility criteria, and search strategy
3. Study selection	Selection of sources for evidence purpose
4. Charting data	Process of data charting, data items, and critical appraisal
5. Collating, summarizing, and reporting findings	Summary of results

**Table 2:** Grey literature search strategy.

Source	Commentary
**Gray literature database**	GreyLit OpenGrey via DANS EASY.
**Gray and published literature database**	PubMed, Scopus, PsychInfo, and Web of Science
**Theses/dissertations**	Proquest Dissertations and Theses Global WorldCat Dissertations and Theses
**Internet search engines**	Google and Google Scholar

**Table 3:** The examples of the search strategy that will be used to generate the articles to review for the research question.

S/N	Database	Search Term	Customization
1	Pubmed	(((education* OR literacy* OR “educational attainment” OR “knowledge about TB/HIV”) OR (economic stability OR poverty* OR “income level” OR “economic status” OR employment* OR “financing health”) OR (neighborhood* OR environment* OR housing* OR sanitation* OR “clean water” OR urbanization* OR “rural health”) OR (“health care” OR “access to health services” OR “health infrastructure” OR “health policy” OR “healthcare delivery” OR “quality of care”) OR (conflict* OR war* OR displacement* OR “refugee health” OR “conflict zones” OR security*) OR (“food stability” OR nutrition* OR “food security” OR malnutrition* OR diet*)) AND (HIV* OR “HIV/AIDS” OR “Human Immunodeficiency Virus”) AND (TB* OR Tuberculosis) AND (comorbidity* OR cooccurring* OR coexistent*)) AND (SSA* OR “Sub-Saharan Africa”)) AND (adult* OR “young adult*” OR “adolescent*” OR age(18-*)))	English; 1 January 2014–15 December 2022
2	WOS	(((education* OR literacy* OR “educational attainment” OR “knowledge about TB/HIV”) OR (economic stability OR poverty* OR “income level” OR “economic status” OR employment* OR “financing health”) OR (neighborhood* OR environment* OR housing* OR sanitation* OR “clean water” OR urbanization* OR “rural health”) OR (“health care” OR “access to health services” OR “health infrastructure” OR “health policy” OR “healthcare delivery” OR “quality of care”) OR (conflict* OR war* OR displacement* OR “refugee health” OR “conflict zones” OR security*) OR (“food stability” OR nutrition* OR “food security” OR malnutrition* OR diet*)) AND (HIV* OR “HIV/AIDS” OR “Human Immunodeficiency Virus”) AND (TB* OR Tuberculosis) AND (comorbidity* OR cooccurring* OR coexistent*)) AND (SSA* OR “Sub-Saharan Africa”)) AND (adult* OR “young adult*” OR “adolescent*” OR age(18-*)))	English; 1 January 2014–15 December 2022
3	SCOPUS	(TITLE-ABS-KEY ((“education*” OR “literacy*” OR “educational attainment” OR “knowledge about TB/HIV”) OR (“economic stability” OR “poverty*” OR “income level” OR “economic status” OR “employment*” OR “financing health”) OR (“neighborhood*” OR “environment*” OR “housing*” OR “sanitation*” OR “clean water” OR “urbanization*” OR “rural health”) OR (“health care” OR “access to health services” OR “health infrastructure” OR “health policy” OR “healthcare delivery” OR “quality of care”) OR (“conflict*” OR “war*” OR “displacement*” OR “refugee health” OR “conflict zones” OR “security*”) OR (“food stability” OR “nutrition*” OR “food security” OR “malnutrition*” OR “diet*”)) AND (“HIV*” OR “HIV/AIDS*” OR “Human Immunodeficiency Virus”) AND (“TB*” OR “Tuberculosis”) AND (“comorbidity*” OR “cooccurring*” OR “coexistent*”)) AND (“SSA*” OR “Sub-Saharan Africa”) AND (“adult*” OR “young adult*” OR “adolescent*” OR AGE(18-*))).	English; 1 January 2014–15 December 2022
4	PsychInfo	((SU (“education*” OR “literacy*” OR “educational attainment” OR “knowledge about TB/HIV” OR “economic stability” OR “poverty*” OR “income level” OR “economic status” OR “employment*” OR “financing health” OR “neighborhood*” OR “environment*” OR “housing*” OR “sanitation*” OR “clean water” OR “urbanization*” OR “rural health” OR “health care” OR “access to health services” OR “health infrastructure” OR “health policy” OR “healthcare delivery” OR “quality of care” OR “conflict*” OR “war*” OR “displacement*” OR “refugee health” OR “conflict zones” OR “security*” OR “food stability” OR “nutrition*” OR “food security” OR “malnutrition*” OR “diet*”) AND (AB (“HIV*” OR “HIV/AIDS*”) OR TI (“Human Immunodeficiency Virus*”)) AND AB(“TB*” OR SU (“Tuberculosis”)) AND (TI (“comorbidity*” OR AB (“cooccurring*”) OR SU (“coexistent*”)) AND (TI (“SSA*”) OR AB (“Sub-Saharan Africa”) AND AB (“young adult*” OR “adolescent*” OR AGE(18-*)))	English; 1 January 2014–15 December 2022
5	Google Scholar	(“education” OR “literacy” OR “educational attainment” OR “knowledge about TB/HIV” OR “economic stability” OR “poverty” OR “income level” OR “economic status” OR “employment” OR “financing health” OR “neighborhood” OR “environment” OR “housing” OR “sanitation” OR “clean water” OR “urbanization” OR “rural health” OR “health care” OR “access to health services” OR “health infrastructure” OR “health policy” OR “healthcare delivery” OR “quality of care” OR “conflict” OR “war” OR “displacement” OR “refugee health” OR “conflict zones” OR “security” OR “food stability” OR “nutrition” OR “food security” OR “malnutrition” OR “diet”) AND (“HIV” OR “HIV/AIDS” OR “Human Immunodeficiency Virus”) AND (“TB” OR “Tuberculosis”) AND (“comorbidity” OR “cooccurring” OR “coexistent”) AND (“SSA” OR “Sub-Saharan Africa”) AND (“adult” OR “young adult” OR “adolescent” OR “age 18 and above”)	English; 1 January 2014–15 December 2022

**Table 4:** Inclusion and exclusion criteria for the scoping review.

S/N	Category	Inclusion Criteria	Exclusion Criteria
1	Language	English	
2	Publication Date Range	January 2014 to December 2022	Before 2014
3	Participant Age	Adolescent 18 and above	Less than 18
4	Population category	HIV/TV Patients	Participants identify as having TB/HIV comorbidity
5	Studies	Social determinants of HIV/TB	Studies on social determinants of HIV/TB co-morbidity
6	Study Design	All designs of primary, secondary, and peer-reviewed studies including randomized controlled trials, intervention studies (including quasi-experimental designs), and qualitative and quantitative studies	Meta-analyses, systematic reviews, and scoping reviews
	Publication date: January 2014 to December 2022. Language: English. Social determinants of TB/HIV Comorbidity		
